# Targeting HIF-1α promotes ferroptosis and boosts antitumor immunity in MSS colorectal cancer

**DOI:** 10.1016/j.redox.2026.104151

**Published:** 2026-04-01

**Authors:** Zhiying Yang, Rui Ma, Weili Wu, Ying Shi, You Chen, Xiaotong Luo, Kai Li, Liangcai Wu, Bo Wang, Boyu Zhang, Ping Yuan

**Affiliations:** a Guangdong Institute of Gastroenterology, Guangzhou, China; b Department of General Surgery, The Sixth Affiliated Hospital, Sun Yat-sen University, Guangzhou, China; c Guangdong Provincial Key Laboratory of Colorectal and Pelvic Floor Disease, The Sixth Affiliated Hospital, Sun Yat-sen University, Guangzhou, China; d Biomedical Innovation Center, The Sixth Affiliated Hospital, Sun Yat-sen University, Guangzhou, China; e Department of Medical Oncology, The Seventh Affiliated Hospital, Sun Yat-sen University, Shenzhen, China; f People's Hospital of Pingshan, Shenzhen, China; g Pingshan Hospital, Southern Medical University, Shenzhen, China; h School of Life Sciences, Sun Yat-sen University, Guangzhou, China; i Department of Dermatology, Heyou Hospital, Shunde District, Foshan, China

**Keywords:** Microsatellite stable, Colorectal cancer, Ferroptosis, HIF-1α

## Abstract

The lack of effective therapeutic options available for microsatellite stable (MSS) colorectal cancer (CRC) remains a significant clinical challenge. Interestingly, chemotherapy-resistant cancer cells can be induced to undergo ferroptosis, prompting our investigation into RSL3, a potent ferroptosis inducer, in MSS CRC cells. Our findings revealed that while RSL3 suppressed the growth of MSS CRC cells, a subset displayed resistance. Single-cell sequencing uncovered an aberrant activation of hypoxia pathways in RSL3-resistant MSS CRC cells. Inhibiting HIF-1α, the key transcription factor driving hypoxia signaling, restored RSL3 sensitivity in these resistant cells; moreover, this sensitivity was attenuated upon HIF-1α overexpression. Chromatin immunoprecipitation assays further demonstrated that in RSL3-resistant cells, HIF-1α was enriched at the promoter of P4HA1, a gene implicated in ferroptosis resistance, thereby enhancing its expression. Additionally, *in vivo* experiments using syngeneic transplantation of CT26 cells in mice revealed that combining RSL3 with an HIF-1α inhibitor markedly enhanced tumor suppression and metastasis prevention, concomitant with increased intratumoral infiltration of CD8^+^ T cells and CD86^+^ macrophages. Notably, the combination enhanced the antitumor response of anti-PD1, a treatment otherwise ineffective on this tumor. These findings suggest that targeting HIF-1α represents a promising therapeutic strategy when used in conjunction with a ferroptosis inducer for the treatment of MSS CRC.

## Graphical abstract

**Figure:**
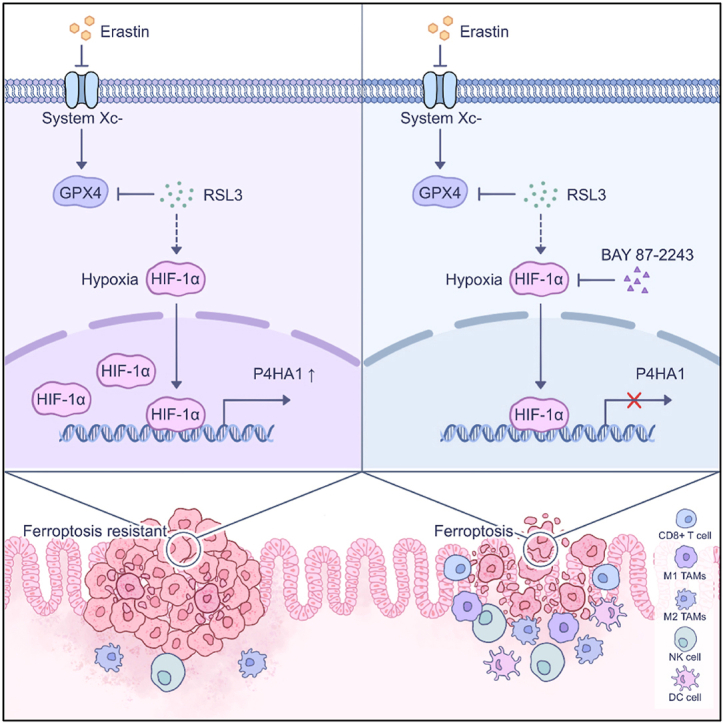

## 1 Introduction

Colorectal cancer (CRC) remains a leading cause of cancer mortality worldwide, with microsatellite stable (MSS) CRC representing the predominant subtype, accounting for approximately 95 % of metastatic cases [1,2]. Unlike high microsatellite instability (MSI-H) tumors, MSS CRC features an intact DNA mismatch repair system and a low tumor mutational burden (TMB), which consequently limits neoantigen presentation [3]. Additionally, MSS CRC demonstrates notably reduced T lymphocyte infiltration and increased accumulation of immunosuppressive cells, including myeloid-derived suppressor cells (MDSCs) [1,4]. These features render MSS CRC largely refractory to immune checkpoint blockade (ICB) therapy, which has revolutionized treatment outcomes for their MSI-H counterparts. Conventional chemotherapeutic agents that induce apoptosis, such as fluorouracil and oxaliplatin, demonstrate limited effectiveness due to the intact DNA repair mechanisms present in MSS CRC [5]. This therapeutic resistance highlights the urgent need for alternative treatment strategies that can circumvent apoptosis-dependent cell death pathways.

Ferroptosis, a type of programmed cell death that relies on iron and involves the buildup of lipid peroxides, has become an attractive therapeutic strategy for treatment-resistant cancers [6,7,8]. Recent research indicates that cancer cells resistant to standard chemotherapy and targeted therapies can undergo ferroptosis when exposed to ferroptosis inducers such as the GPX4 inhibitor RSL3 and the SLC7A11 inhibitor Erastin. These findings suggest that ferroptosis induction may offer therapeutic potential for malignancies unresponsive to conventional treatments [9,10,11].

Moreover, studies have demonstrated that the induction of ferroptosis can alter the immune microenvironment by increasing the immunogenicity of tumor and inhibiting the immunosuppressive cells. Ferroptotic cells could release damage-associated molecular patterns (DAMPs), which enhance tumor immunogenicity, activate dendritic cells, and promote T cell response [12,13]. On the other hand, effective CD8^+^ T cells produce IFN-γ to downregulate SLC7A11 expression in tumor cells, leading to ferroptosis and thereby amplifying antitumoral responses [14]. Furthermore, the tumor microenvironment (TME) is often dominated by M2 tumor-associated macrophages (TAMs). Targeting ferroptosis in tumor cells can induce the polarization from M2 to M1, improving ICBs’ therapeutic efficacy [15,16]. In summary, triggering ferroptosis in tumor cells may elicit a vaccine-like immune response, ultimately enhancing the efficacy of antitumor effects.

However, our investigation revealed that while RSL3 suppressed the growth of MSS CRC cells, a subset exhibited resistance to ferroptosis, and the mechanisms were incompletely understood. Single-cell sequencing revealed that the hypoxia signaling pathway was excessively activated in the ferroptosis-resistant cells. We hypothesized that the activation of the hypoxia signaling pathway might confer resistance to ferroptosis in MSS CRC cells.

Hypoxia-inducible factor 1α (HIF-1α) serves as a central regulatory hub mediating cellular adaptation to hypoxic microenvironments through both oxygen-dependent degradation mechanisms and oxygen-independent oncogenic signaling pathways [17]. Beyond its role as a master regulator of hypoxia response, HIF-1α also plays a pivotal role in driving glycolytic metabolism by activating genes involved in glycolysis [18,19]. Hence, HIF-1α activation has been implicated in various aspects of cancer biology, including therapy resistance, metastasis, and immune evasion [20,21,22,23]. Indeed, suppression of HIF-1α reduced the resistance of MSS CRC cells to ferroptosis, while overexpression of HIF-1α enhanced this resistance. These findings confirm the essential role of HIF-1α in mediating ferroptosis resistance.

Moreover, we generated ferroptosis-resistant MSS CT26 cells through chronic, step-wise exposure to escalating doses of RSL3 and found increased nuclear localization of HIF-1α in the acquired cells. Chromatin immunoprecipitation (ChIP) assays further indicated the increased enrichment of HIF-1α at the promoter region of P4HA1 in ferroptosis-resistant cells. P4HA1, or prolyl 4-hydroxylase alpha subunit 1, encodes a critical enzyme involved in the hydroxylation of proline residues within collagen, a process vital for stabilizing collagen structure and maintaining connective tissue integrity [24]. P4HA1 has been identified as a significant regulator of cancer progression, disrupting the tricarboxylic acid (TCA) cycle through alterations in α-ketoglutarate and succinate metabolism. This disruption leads to mitochondrial dysfunction and facilitates metabolic adaptations that favor tumor growth [25]. Recent research has also highlighted P4HA1's role as a pivotal regulator of ferroptosis resistance in nasopharyngeal carcinoma, ultimately leading to decreased lipid peroxidation, an essential characteristic of ferroptosis [26].

In this study, we revealed that the HIF-1α-P4HA1 axis enhanced the resistance of MSS CRC cells against ferroptosis. Subsequent *in vivo* experiments showed that simultaneous targeting of HIF-1α and ferroptosis effectively diminished tumor growth and liver metastasis in MSS CRC. Interestingly, single-cell sequencing analysis of residual tumors after treatment showed that the dual targeting strategy increased infiltration of CD8^+^ T cells and CD86^+^ macrophages. This finding indicates a transition of the MSS CRC tumor from an immunologically ‘cold’ to a ‘hot’ tumor status. These observations establish a mechanistic rationale for synergistic therapeutic approaches that simultaneously disrupt ferroptosis resistance and modulate the immunosuppressive TME.

## 2 Materials and methods

### 2.1 Cell culture

The human MSS CRC cell lines SW480, HT-29, and WiDr, human HEK293T, and mouse MSS CRC cell line CT26 were obtained from American Type Culture Collection (ATCC). All cell lines were confirmed to have no mycoplasma contamination and incubated at 37 °C with 5 % (vol/vol) CO_2_. SW480, HT-29, WiDr, and CT26 were cultured in RPMI 1640 medium supplemented with 10 % fetal bovine serum (FBS) and 1 % penicillin-streptomycin. HEK293T cells were maintained in Dulbecco's modified Eagle's medium (DMEM) supplemented with 10 % FBS and 1 % penicillin-streptomycin.

### 2.2 Microwell-seq

Microwell-seq was conducted as previously described [27]. Briefly, cells or tissues were trypsinized to obtain a single-cell suspension. 1 × 10^5^ cells of each sample were added to an agarose microwell plate, followed by supplementation with oligonucleotide-conjugated magnetic beads. Then, cells were lysed and subjected to reverse transcription. The DNA library was amplified, screened, and subsequently sequenced using the Illumina HiSeq X Ten system.

For Microwell-seq data analysis, raw data were processed using the sequence filtering module bbduk2 from the BBTools suite. A stepwise filtering strategy was applied to remove low-quality data containing ‘CGACTCACTACAGGG’, ‘TCGGTGACACGATCG’, and ‘TTTTTTTTTTTT’ sequences. Following initial data cleaning, Fastq ToSam from the Picard toolkit was employed to convert Fastq-formatted sequencing data into standardized alignment formats. The data were further compressed into binary BAM files to optimize storage efficiency. Subsequent processing of BAM files was conducted using the Drop-seq pipeline. This process culminated in the generation of a quantitative gene expression matrix.

Next, we analyzed data using the Seurat R package. Cells with UMI counts below 500 were excluded for downstream bioinformatic analyses. A dual filtering strategy based on Seurat's built-in quality standards was implemented: cells with fewer than 200 genes or more than 8000 genes were discarded, while those exhibiting mitochondrial gene expression exceeding 10 % were also removed. Finally, t-distributed stochastic neighbor embedding (t-SNE) dimensionality reduction was applied to visualize and resolve the distribution of cell subpopulations. Differentially expressed genes for each cell cluster are shown in Supplementary Table 1-3.

### 2.3 RNA isolation and quantitative real-time PCR (qPCR)

After treatment with indicated conditions, cells were lysed in Trizol (Invitrogen, cat# 15596-026) for total RNA extraction. RNA was then reverse-transcribed into cDNA using *EasyScript®* First-Strand cDNA Synthesis SuperMix (TransGen, cat# AE301-03) according to the manufacturer's instructions. qPCR was performed using *PerfectStart*® Green qPCR SuperMix (TransGen, cat# AQ601-02). The mRNA expression levels of the target genes were normalized against β-actin, the endogenous reference. All assays were performed with three independent replicates. The primer sequences of targeted genes are shown in Supplementary Table 4.

### 2.4 Western blotting analysis

Cells were rinsed using ice-cold PBS and lysed in lysis buffer supplemented with both phosphatase and protease inhibitors. Protein samples were subjected to SDS-polyacrylamide gel electrophoresis and subsequently transferred electrophoretically onto PVDF membranes. The antibodies used in this research were as follow: HIF-1α (Proteintech, cat# 20960-1-AP, 1:2000), P4HA1 (Proteintech, cat# 12658-1-AP, 1:2000), GPX4 (Proteintech, cat# 67763-1-Ig, 1:1000), β-actin (Santa Cruz, cat# sc-47778, 1:1000), α-tubulin (Proteintech, cat# 66031-1-Ig, 1:1000), Histone 3 (Cell Signaling Technology, cat# 4499s, 1:1000), 4-HNE (Abcam, cat# ab46545, 1:1000).

### 2.5 DNA constructs, transfection, and lentivirus production

HIF-1α cDNA was amplified from HEK293T by PCR with the following primers: cl-pLenti-HIF1α-F: ATGGAGGGCGCCGGCG; cl-pLenti-HIF1α-R: TCAGTTAACTTGATCCAAAGCTCTGA. HIF-1α cDNA was subsequently sub-cloned into the PB-TRE-EGFP-EF1α-rtTA vector (Addgene) to generate a doxycycline-inducible HIF-1α overexpression construct. For the doxycycline-inducible HIF-1α knockdown, short-hairpin RNAs targeting HIF-1α were designed: shHIF-1α#1: GTGATGAAAGAATTACCGAAT; shHIF-1α#2: TGCTCTTTGTGGTTGGATCTA. Targeted sequences were integrated into the pLKO-Tet-On vector (Addgene). All constructs generated were verified by DNA sequencing.

For the establishment of stable cell lines, HEK293T cells were plated onto 10 cm^2^ dishes. After 60 % confluence, cells were co-transfected with psPAX2, pMD2.G, and the indicated construct in the ratio of 1:1:2. The supernatant containing lentivirus was collected after 48 and 72 h of transfection. The MSS CRC cells were infected with lentivirus containing 8 μg/ml polybrene (Millipore, TR-1003-G) and further selected with puromycin. To induce HIF-1α knockdown or overexpression, 200 ng/ml doxycycline (Meilunbio, cat# MB1088-1) was added to the culture medium for 72 h.

### 2.6 Cell viability assay

Cells were seeded in 96-well plates with or without doxycycline. After 24 h, cells were treated with RSL3 (Aladdin, cat# R302648), Erastin (TargetMol, cat# T1765), or BAY 87-2243 (TargetMol, cat# T2488) for 48 h. Then, cells were incubated with 10 % CCK-8 for 3 h. Absorbance at 450 nm was measured using a microplate reader. The half-maximal inhibitory concentration (IC_50_) for each compound was calculated using GraphPad Prism 8.0.

### 2.7 DCFH-DA oxidation and lipid peroxidation assays

DCFH-DA fluorescence probe (Beyotime, cat# S0033 M) was used to assess intracellular oxidative stress levels. Briefly, cells were seeded in 6-well plates and treated with or without doxycycline as indicated. After treatment, the culture medium was aspirated and replaced with DCFH-DA at a final concentration of 10 μM, and cells were incubated at 37 °C in the dark for 30 min. Cells were then harvested, washed and resuspended in PBS for flow cytometry analysis. Fluorescence was acquired in the FITC channel (excitation/emission: 488/510 nm). Mean fluorescence intensity of the oxidized DCFH-DA product was recorded as a measure of intracellular oxidative stress.

Lipid peroxidation was assessed in parallel using the fluorescent probe C11-BODIPY 581/591 (Invitrogen, cat# D3861). Following the same seeding and treatment conditions described above, cells were labeled with C11-BODIPY 581/591 according to the manufacturer's instructions. Upon harvesting, samples were analyzed via flow cytometry. The oxidation-dependent shift in fluorescence emission from red (∼590 nm) in the reduced form to green (∼510 nm) upon oxidation was measured in the FITC channel. The mean fluorescence intensity of the oxidized C11-BODIPY signal was calculated and compared across experimental groups.

### 2.8 Malondialdehyde (MDA) assay

Cells were collected after treatment at a minimum of 1 × 10^6^ cells per experimental group. Cells were washed once with PBS and lysed in 100 μl RIPA lysis buffer containing protease inhibitors on ice for 30 min. Then cell lysate supernatant was harvested by centrifugation. Subsequently, 100 μl supernatant was transferred to a new 1.5 ml tube, mixed with 200 μl thiobarbituric acid solution (Beyotime, cat# S0131S), and incubated at 100 °C for 1.5 h. A blank control was prepared in parallel using 100 μl RIPA lysis buffer under the same conditions. After incubation, samples were cooled in an ice-water bath and centrifuged at 1000×*g* for 10 min. Finally, 200 μl supernatant from each sample was measured at 532 nm using a microplate reader.

### 2.9 Glutathione (GSH) measurement

GSH level was detected using the GSH and GSSG Assay Kit (Beyotime, cat# S0053) according to the manufacturer's instructions. In briefly, cells were collected and lysed in protein removal reagent using a sonicator. After centrifugation, cell lysate supernatant was obtained for total Glutathione and GSSG assays. GSH production was determined by formula: GSH = total Glutathione - GSSG × 2.

### 2.10 Sphere formation assay

SW480, HT-29, or WiDr were treated with either DMSO or RSL3 for 48 h, and then living cells were collected using trypan blue and seeded at a density of 1 × 10^3^ cells per well into low-attachment 24-well plates. Afterwards, cells were cultured in DMEM/F12 supplemented with 40 ng/ml EGF, 20 ng/ml bFGF, and B27 (1:50 dilution). The number of tumor spheres larger than 70 μm in diameter was counted to evaluate self-renewal capacity.

### 2.11 In vivo studies

All Balb/c mice used in this study were obtained from Guangdong GemPharmatech Co., Ltd. Animal procedures were approved by the Ethical and Welfare Committee of the Sixth Affiliated Hospital of Sun Yat-sen University.

For the subcutaneous tumor model, 5 × 10^5^ parental CT26 or RSL3-resistant CT26 cells (Re-CT26) were collected in PBS and subcutaneously inoculated into the right flank of mice. Mouse body weight was recorded from the day of implantation, and tumor size was measured every 2 days thereafter.

To evaluate the efficacy of different treatments, mice were subcutaneously injected with 5 × 10^5^ CT26 cells. After tumor size reach 50 mm^2^, mice were randomly separated into the following groups and treated accordingly: control group (vehicle), RSL3 group (80 mg/kg, intraperitoneally injection, three times per week), BAY 87-2243 group (3 mg/kg, oral gavage, daily), anti-PD1 (Selleck, cat# A2122; 10 mg/kg, intraperitoneally injection, twice per week), RSL3+anti-PD1 (RSL3: 80 mg/kg, intraperitoneally injection, three times per week; anti-PD1: 10 mg/kg, intraperitoneally injection, twice per week), BAY 87-2243+anti-PD1 (BAY 87-2243: 3 mg/kg, oral gavage, daily; anti-PD1: 10 mg/kg, intraperitoneally injection, twice per week), RSL3+BAY 87-2243 (RSL3: 80 mg/kg, intraperitoneally injection, three times per week; BAY 87-2243: 3 mg/kg, oral gavage, daily), RSL3+BAY 87-2243+anti-PD1 (RSL3: 80 mg/kg, intraperitoneally injection, three times per week; BAY 87-2243: 3 mg/kg, oral gavage, daily; anti-PD1: 10 mg/kg, intraperitoneally injection, twice per week). Mouse body weight was monitored from the day of implantation, and tumor size was measured every two days.

For the liver metastasis tumor model, 5 × 10^5^ parental CT26 or Re-CT26 cells were intrasplenically transplanted into Balb/c mice. Subsequent monitoring of liver metastasis was performed at 4-day intervals using an IVIS Spectrum In vivo Imaging System (PerkinElmer).

To evaluate the efficacy of different treatments, 5 × 10^5^ CT26 cells were intrasplenically transplanted into balb/c mice. Mice were randomly separated into four groups with indicated treatment in second day: control group (vehicle, intraperitoneally injection), RSL3 group (80 mg/kg, intraperitoneally injection, three times per week), BAY 87-2243 group (3 mg/kg, oral gavage, daily), combination treatment group (80 mg/kg, intraperitoneally injection, three times per week; 3 mg/kg, oral gavage, daily). Mouse body weight was measured, and liver metastasis was monitored using an *in vivo* imaging system.

### 2.12 Immunohistochemical staining

Immunohistochemical staining was conducted as previously described [28]. Paraffin-embedded tissue sections were deparaffinized in xylene and rehydrated through a graded ethanol series. Antigen retrieval was carried out with sodium citrate buffer in a high-pressure heating apparatus. After cooling to room temperature, the sections were treated with 3 % hydrogen peroxide for 10 min and blocked with goat serum for 1 h. The slices were then incubated with primary antibodies at 4 °C overnight. The following day, the sections were exposed to secondary antibodies for 15 min at room temperature and subsequently visualized using diaminobenzidine (DAB). The antibodies used here were as follow: Ki67 (Abcam, cat# ab15580, 1:300), 4-HNE (Abcam, cat# ab46545, 1:100), CD8 (AiFang biological, cat# 98941S, 1:200), CD86 (AiFang biological, cat# 13395-1-AP, 1:200).

### 2.13 Bioinformatic analysis

The association between gene expression and survival in CRC was interrogated using the KM Plotter database (http://kmplot.com/analysis/index.p). On the platform homepage, ‘Colon cancer’ was selected as the cancer type, and the gene symbol or Affymetrix probe ID was entered to evaluate progression-free survival (PFS). Tumor subtype was selected with ‘stable or low’ for HIF-1α analysis and ‘stable’ for P4HA1 analysis. Using the median expression level as the cutoff, patients were stratified into high and low expression groups. The differences of Kaplan-Meier survival curves between groups were assessed using the log-rank test, with a significance threshold set at a *P* value lower than 0.05. Hazard ratios (HR) and corresponding confidence intervals were also calculated.

Correlation between HIF-1α and P4HA1 was analyzed using the TIMER database (https://cistrome.shinyapps.io/timer/). For the tumor type ‘Colon adenocarcinoma’, correlation analysis of gene expression levels was performed using the default Spearman's rank correlation coefficient to assess monotonic relationships between variables.

### 2.14 ChIP qPCR

ChIP assays were carried out as previously described [29]. Re-CT26 and parental CT26 cells were fixed with 1 % formaldehyde for 20 min at room temperature and then quenched with 0.125 M glycine for 5 min. After washing with PBS, cells were harvested in ChIP lysis buffer containing protease inhibitors. Chromatin was sheared using a sonicator. Cleared lysates were immunoprecipitated overnight at 4 °C with 2 μg of HIF-1α antibody (Proteintech, cat# 20960-1-AP) or control IgG in the presence of 20 μl Magna ChIP™ Protein A + G Magnetic Beads (Millipore, cat# 16–663). The beads were sequentially washed with low-salt wash buffer, high-salt wash buffer, LiCl buffer, and TE buffer. Bound chromatin was eluted in 50 μl elution buffer containing 5 μl proteinase K (20 mg/ml) and incubated overnight at 65 °C. DNA was purified using a commercial PCR purification kit (Omega, cat# D2500-02) and analyzed by qPCR.

### 2.15 Statistical analysis

All data are presented as mean ± standard deviation (SD) except single-cell analysis. Data visualization and statistical analysis were performed using GraphPad Prism version 8.0. Comparisons between two groups were conducted using the *t*-test or two-way ANOVA; multiple-group comparisons were carried out by one-way ANOVA followed by Tukey's multiple comparison test or Dunnett's post hoc test, unless otherwise stated in the figure legend. A p-value less than 0.05 was considered statistically significant.

## 3 Results

### 3.1 Single-cell transcriptome analysis revealed a ferroptosis-resistant subset in MSS CRC

To identify the effects of the ferroptosis inducer in MSS CRC, HT-29, SW480, or WiDr cells were treated with RSL3 or Erastin. Cell death was induced by both RSL3 and Erastin at the indicated concentrations (Supplementary Fig. S1A). GPX4 protein was down-regulated after 48 h of RSL3 or Erastin treatment (Supplementary Fig. S1B). To further confirm ferroptotic induction, we assessed oxidative stress and lipid peroxidation, two defining biochemical hallmarks of ferroptosis, by flow cytometry. Treatment with either RSL3 or Erastin markedly elevated DCFH-DA oxidation, reflecting increased intracellular oxidative stress (Supplementary Fig. S1C and S1D) and significantly increased C11-BODIPY oxidation, indicative of enhanced lipid peroxidation (Supplementary Fig. S1E and S1F), collectively confirming that both agents effectively trigger ferroptosis in MSS CRC cells.

**Fig. 1:**
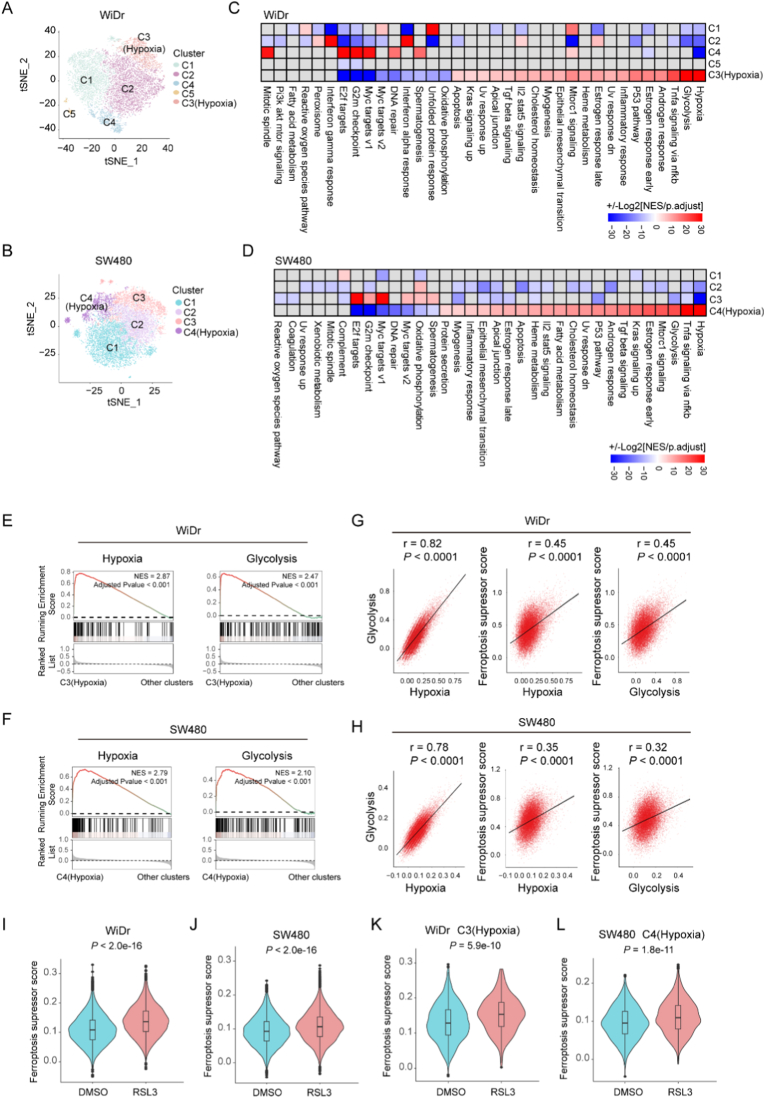
A hypoxia-characteristic cluster identified by Microwell-seq exhibited pronounced ferroptosis resistance in MSS CRC cells. (A and B) t-distributed stochastic neighbor embedding (t-SNE) plot of Microwell-seq analysis based on gene expressions of SW480 and WiDr. (C and D) Comparative gene set enrichment analysis (GSEA) of signaling pathways in different clusters. The red color represents up-regulation and blue represents down-regulation, calculated with the formula: ± Log2|NES/p.adjust|. Grey color represents no enrichment in the indicated pathway. NES: normalized enrichment score. (E and F) GSEA analysis showed the indicated pathway activity between the hypoxia cluster and other clusters. (G and H) Correlation analysis of hypoxia scores, glycolysis scores, and ferroptosis suppressor scores in WiDr and SW480 cells was performed using Pearson's method. (I and J) The ferroptosis suppressor score of SW480 and WiDr with DMSO or RSL3 treatment was analyzed by AddModuleScore tool. *P* values were calculated by Wilcox.test. (K and L) The ferroptosis suppressor score of hypoxia cluster in SW480 and WiDr treated with DMSO or RSL3 was shown. *P* values were calculated by Wilcox.test.

To further understand the alteration of MSS CRC cells under ferroptosis inducer treatment, we conducted single-cell transcriptome analysis. WiDr or SW480 cells were treated with DMSO or RSL3 for 48 h, and cells were collected for Microwell-seq. Based on the gene-expression profiles, we stratified WiDr and SW480 cells into different subclusters (Fig. 1A and B). Notably, we identified a similar subtype characterized by abnormal hypoxic and glycolytic activation that was prominently distinguished from other clusters (Fig. 1C and D). Gene set enrichment analysis (GSEA) across all subclusters showed diverse signaling pathways. The normalized enrichment score (NES) of hypoxia and glycolysis pathways in the hypoxia cluster was higher than 2.0 (Fig. 1E and F). Further analysis revealed strong positive correlations among hypoxia scores, glycolysis scores, and ferroptosis resistance scores at the single-cell level: cells with elevated hypoxia scores exhibited correspondingly higher glycolysis scores and greater ferroptosis resistance, while cells with higher glycolysis scores similarly displayed enhanced ferroptosis resistance scores (Fig. 1G and H). In addition, after treatment with RSL3, WiDr and SW480 exhibited increased ferroptosis resistance (Fig. 1I and J). Furthermore, the cluster characterized with high hypoxic activity showed enhanced resistance to ferroptosis after RSL3 treatment (Fig. 1K and L). These data demonstrated the presence of a hypoxia-active subpopulation within MSS CRC cells that displayed robust ferroptosis resistance.

### 3.2 HIF-1α overexpression was associated with poor prognosis in MSS CRC

**Fig. 2:**
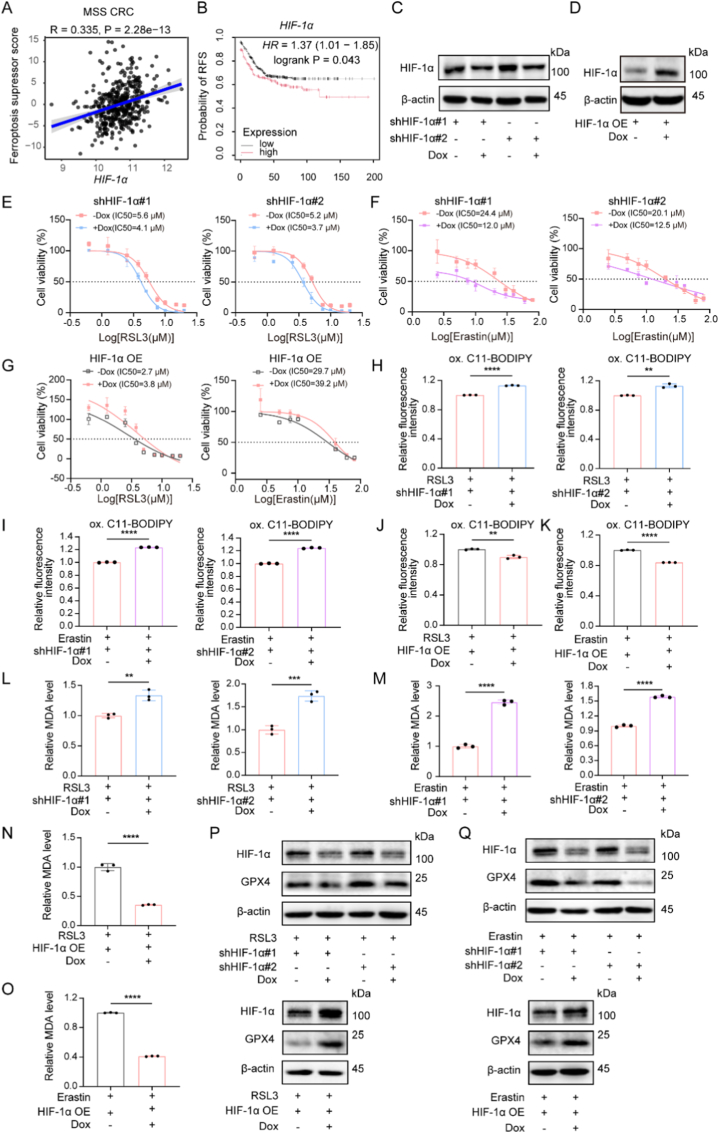
HIF-1α promoted ferroptosis resistance and was associated with poor prognosis in MSS CRC. (A) The correlation between *HIF-1α* level and ferroptosis suppressor score was analyzed using Spearman's method. (B) Kaplan-Meier plots of RFS in microsatellite stable/low unstable colon cancer patients, stratified by *HIF-1α* gene expression. RFS: relapse-free survival. (C) Western blot validation of HIF-1α knockdown efficiency in SW480 cells. HIF-1α knockdown was induced by doxycycline treatment. Dox: doxycycline. (D) Western blot validation of HIF-1ɑ overexpression (OE) efficiency in SW480 cells. HIF-1α overexpression was induced by doxycycline treatment. Dox: doxycycline. (E and F) Cell viability of SW480 cells, with or without HIF-1α knockdown, was assessed using the CCK-8 assay after treating with graded concentrations of RSL3 or Erastin. (G) Cell viability of SW480 cells, with or without HIF-1α overexpression, was shown after treatment with graded concentrations of RSL3 or Erastin. (H) Lipid peroxidation was assessed by flow cytometric detection of oxidized C11-BODIPY 581/591 (FITC channel; excitation/emission: 488/510 nm) after RSL3 treatment. The fluorescence intensity of oxidized C11-BODIPY reflects the lipid peroxidation levels. ox. C11-BODIPY: oxidized C11-BODIPY. (I) Lipid peroxidation levels were detected by flow cytometry after Erastin treatment. (J and K) Lipid peroxidation levels of SW480, with or without HIF-1α overexpression, was detected by flow cytometry after treatment with the indicated concentration of RSL3 or Erastin. (L-O) MDA levels of SW480 cells with or without HIF-1α knockdown or overexpression following treatment with RSL3 or Erastin. (P and Q) Western blot validation of GPX4 protein levels in SW480 cells with or without HIF-1α knockdown or overexpression following treatment with RSL3 or Erastin. Data are presented as means ± SD. ∗*P* < 0.05; ∗∗*P* < 0.01; ∗∗∗*P* < 0.001; ∗∗∗∗*P* < 0.0001. *P* values were calculated by two-tailed *t*-test.

In solid tumors, hypoxia often occurs and is primarily regulated by HIF-1α, a key transcription factor that mediates cellular responses and consequently drives metabolic reprogramming in cancer cells [30]. Further analysis of MSS CRC data from the dataset GSE39582 revealed that higher *HIF-1α* expression positively correlated with ferroptosis suppressor score (Fig. 2A). To explore the role of HIF-1α in MSS CRC, we first performed prognostic analysis using the Kaplan Meier Plotter database. High *HIF-1α* expression was correlated with poor relapse-free survival in microsatellite stable/low microsatellite unstable colon cancer patients (n = 508) (Fig. 2B). Furthermore, the gain and loss of function studies were performed in SW480 cells. shRNA-mediated knockdown and overexpression of HIF-1α were conducted, and their efficiency was validated by Western blot (Fig. 2C–D). HIF-1α knockdown increased cell death induced by either RSL3 or Erastin treatment (Fig. 2E–F). Conversely, overexpression of HIF-1α decreased cell death induced by RSL3 or Erastin in SW480 cells (Fig. 2G). Similarly, knockdown of HIF-1α enhanced intracellular lipid peroxidation accumulation induced by RSL3 or Erastin (Fig. 2H–I), whereas lipid peroxidation levels were suppressed by HIF-1α overexpression in SW480 (Fig. 2J–K). As MDA is a product of lipid peroxidation and a recognized indicator of ferroptosis [31], MDA levels were subsequently assessed. MDA levels were increased after HIF-1α knockdown and decreased after HIF-1α overexpression in the context of RSL3 or Erastin treatment (Fig. 2L-O). In addition, HIF-1α knockdown led to decreased GPX4 after RSL3 or Erastin exposure, while the opposite effects were observed in HIF-1α-overexpressing cells (Fig. 2P–Q). Collectively, these findings demonstrate that HIF-1α promoted ferroptosis resistance in MSS CRC cells.

### 3.3 Ferroptosis-resistant cells, characterized by HIF-1α activation, significantly promoted tumor growth and metastasis

**Fig. 3:**
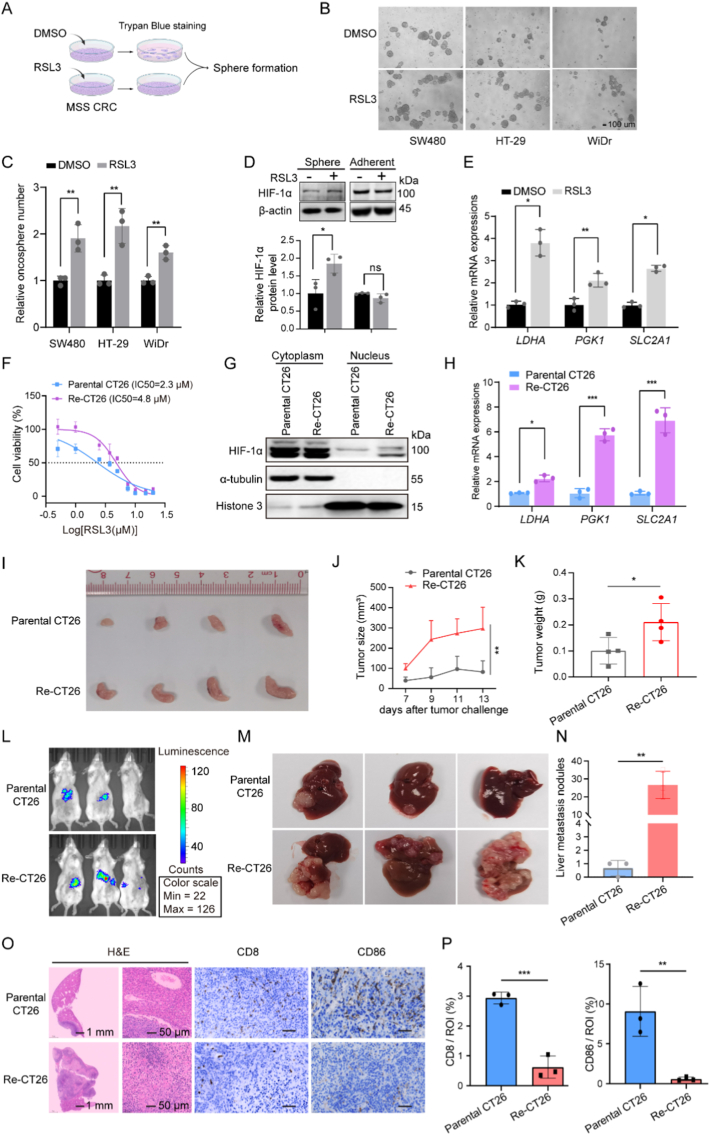
HIF-1α nuclear distribution increased in RSL3-resistant CT26 cells and significantly promoted tumourigenicity and metastasis. (A) The diagram demonstrated the procedure for sphere formation. (B and C) The representative images of sphere formation in MSS CRC cells and quantification analysis of sphere number derived from SW480, HT-29, and WiDr. (D) HIF-1α expression in WiDr was detected by western blotting. (E) qPCR analyzed the indicated gene expression involved in glycolysis in WiDr spheres. (F) Cell viability of parental CT26 and RSL3-resistant CT26 (Re-CT26). (G) Cytoplasm and nuclear HIF-1α expression in CT26. α-tubulin was used as an internal reference for the cytoplasm, and Histone 3 was used as a nuclear reference. (H) qPCR analyzed the indicated gene expression involved in glycolysis in CT26. (I) Image of subcutaneous tumors derived from parental CT26 and Re-CT26. (J) Tumor volume determined by formula: 0.52 × Long × width^2^. (K)Tumor weight of subcutaneous tumors. (L) Bioluminescent images in liver metastatic models. (M) Images of liver metastasis derived from parental CT26 and Re-CT26. (N) Number of liver nodules in liver metastasis models. (O) Representative hematoxylin and eosin (H&E) and immunohistochemical staining images from liver metastasis models. Scale bar: 25 μm. (P) Quantification of immunohistochemical staining results shown in (O). Data are shown as means ± SD. ∗*P* < 0.05; ∗∗*P* < 0.01; ∗∗∗*P* < 0.001; ns: not significant. Two-way ANOVA in (J), others unpaired two-tailed Student's *t*-test.

Single-cell analysis revealed heterogeneity in MSS CRC cells. Given that HIF-1α promoted ferroptosis resistance in MSS CRC cells, we wondered whether the ferroptosis-resistant subpopulation showed enhanced HIF-1α activation and increased survival ability. We pretreated cells with DMSO or RSL3 for 48 h, then the viable cells determined by trypan blue exclusion were collected and further applied to sphere formation assays (Fig. 3A). Results showed that increased sphere formation by RSL3 screening, which indicated the enhanced ability of cell self-renewal in WiDr, HT-29, or SW480 cells (Fig. 3B–C). Moreover, HIF-1α protein level increased in spheres derived from RSL3-treated WiDr but not in adherent cells (Fig. 3D). The mRNA levels of genes regulating glycolysis also showed significant upregulation (Fig. 3E).

To better understand the relevance of HIF-1α to ferroptosis in MSS CRC, we established a cell line, Re-CT26, which was resistant to RSL3 compared with parental CT26 treated with DMSO (Fig. 3F). The nuclear protein level of HIF-1α increased in Re-CT26, indicating the transcriptomic alteration of HIF-1α (Fig. 3G). *LDHA*, *PGK1* and *SLC2A1*, which are involved in glycolysis, were highly amplified in Re-CT26 mimicking the hypoxia cluster in SW480 or WiDr cells (Fig. 3H). To determine the tumor-initiating capacity and metastasis potential, Re-CT26 and parental CT26 cells were implanted into balb/c mice, respectively. Tumor initiated by Re-CT26 grew faster than the DMSO control (Fig. 3I-K). Tumor metastasis was evaluated in intrasplenic injection mouse models. Since CT26 was labeled with luciferase previously, liver metastatic dynamics could be tracked using an IVIS Spectrum In vivo Imaging System. Ferroptosis-resistant Re-CT26 showed higher liver metastatic intensity (Fig. 3L-N), and significantly decreased CD8^+^ T cells and CD86^+^ macrophages infiltration determined by immunostaining in liver metastatic tissues (Fig. 3O–P). These results suggested that HIF-1α driving ferroptosis resistance was essential for tumor malignant progression.

### 3.4 HIF-1α directly regulated P4HA1 to confer ferroptosis resistance

**Fig. 4:**
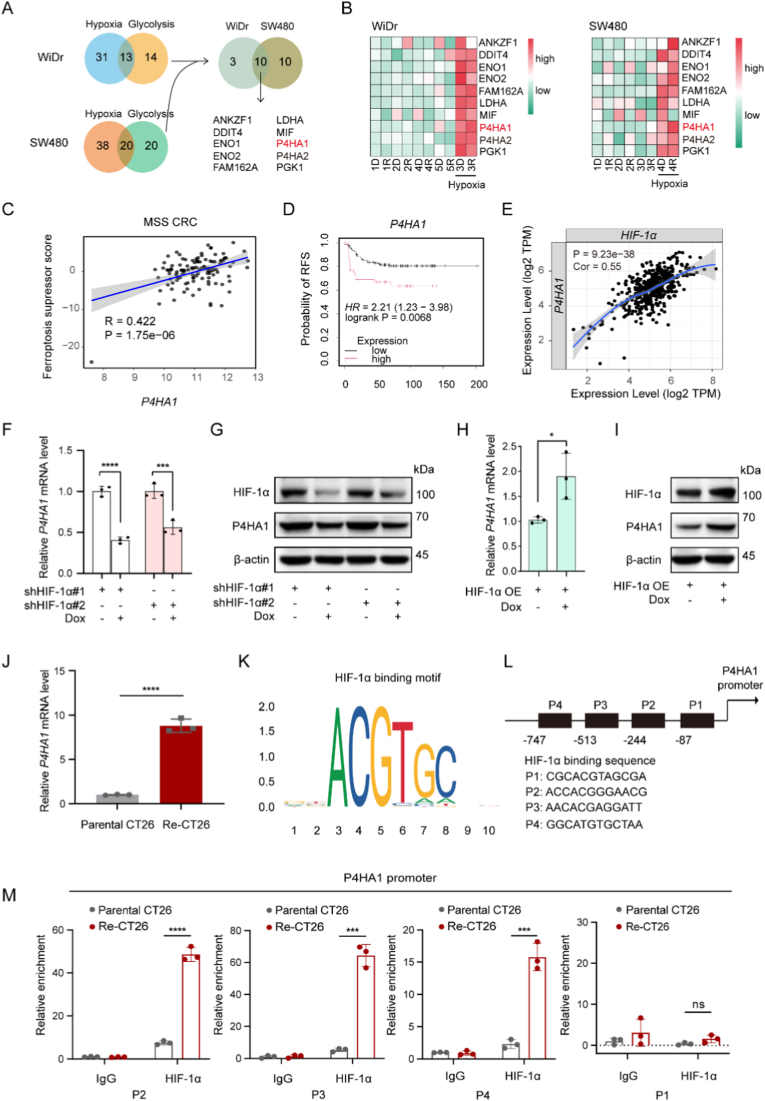
P4HA1 was a major factor regulated by HIF-1α and was enriched in ferroptosis-resistant cells. (A) Venn diagram screening 10 genes commonly induced by hypoxia and the glycolysis pathway in SW480 and WiDr by single-cell sequencing. (B) Gene expression heatmap showed the distribution of genes in different clusters in the presence or absence of RSL3. D: DMSO, R: RSL3. (C) Correlation between ferroptosis suppressor score and P4HA1 in MSS CRC containing 119 patients using Pearson's method. (D) Kaplan-Meier plots of RFS in MSS colon cancer patients according to P4HA1 expression. (E) The correlation between HIF-1α and P4HA1 was evaluated by Spearman's analysis in a colon adenocarcinoma cohort of 457 patients. (F) Relative *P4HA1* mRNA expression after HIF-1α knockdown, detected by qPCR. (G) Relative P4HA1 protein expression after HIF-1α knockdown, detected by Western blot. (H) Relative *P4HA1* mRNA expression after HIF-1α overexpression, detected by qPCR. (I) Relative P4HA1 protein expression after HIF-1α overexpression, detected by Western blot. (J) Relative P4HA1 expression, detected by qPCR. (K) HIF-1α binding motif predicted from JASPAR. (L) The prospective binding site of HIF-1α on the promoter of P4HA1. (M) ChIP assay of HIF-1α and IgG in parental CT26 cells or Re-CT26 cells, followed by qPCR for the binding sequences.

To elucidate the underlying mechanisms of HIF-1α-mediated ferroptosis resistance, we performed single-cell RNA sequencing analysis to identify genes enriched at the intersection of hypoxia and glycolysis pathway in both SW480 and WiDr. Ten overlapped genes were revealed: *ANKZF1*, *DDIT4*, *ENO1*, *ENO2*, *FAM162A*, *LDHA*, *MIF*, *P4HA1*, *P4HA2* and *PGK1* (Fig. 4A). Gene expression analysis demonstrated that *P4HA1*, encoding prolyl 4-hydroxylase subunit alpha 1, was highly induced in hypoxia cluster and further elevated upon RSL3 treatment (Fig. 4B). MSS CRC data analyses from dataset GSE26682 demonstrated that *P4HA1* expression was highly correlated with ferroptosis defense (Fig. 4C). Further analyze showed high *P4HA1* expression was associated with poor patient outcomes in MSS colon cancer (Fig. 4D), confirming its oncogenic role consistent with previous studies [25]. In addition, we found that *HIF-1α* expression was positively related to *P4HA1* level in colon adenocarcinoma (Fig. 4E). To confirm HIF-1α-dependent regulation of P4HA1, we performed knockdown and overexpression experiments. As expected, *P4HA1* expression at both mRNA and protein levels was reduced by HIF-1α knockdown (Fig. 4F–G) and increased by HIF-1α overexpression (Fig. 4H–I). Furthermore, the qPCR assay showed that *P4HA1* expression significantly increased in Re-CT26 cells (Fig. 4J). To validate HIF-1α-mediated transcriptional regulation of P4HA1, we performed ChIP-qPCR in Re-CT26 and parental CT26 cells. The results demonstrated enhanced HIF-1α binding at the P4HA1 promoter region in ferroptosis-resistant Re-CT26 cells compared to parental CT26 cells (Fig. 4K-M). These findings suggested that HIF-1α-mediated transcription of P4HA1 was upregulated in Re-CT26 cells; consistently, enhanced nuclear accumulation of HIF-1α was observed in Re-CT26 cells (Fig. 3G). Consequently, the activation of the HIF-1α-P4HA1 axis may confer ferroptosis resistance in MSS CRC.

### 3.5 Inhibition of HIF-1α enhanced the ferroptosis sensitivity of MSS CRC cells *in vitro*

**Fig. 5:**
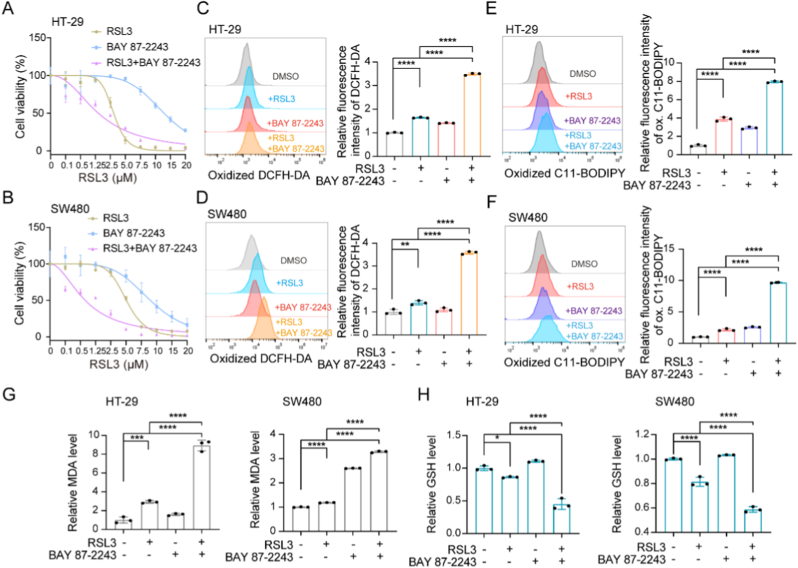
HIF-1α inhibition enhanced ferroptosis inducer sensitivity in MSS CRC cells. (A and B) Cell viability of HT-29 or SW480 treated with RSL3, BAY 87-2243 alone or in combination treatment. (C and D) DCFH-DA oxidation in HT-29 or SW480 treated with RSL3, BAY 87-2243 alone or in combination treatment were quantified using flow cytometry with DCFH-DA probe. (E and F) Lipid peroxidation levels of HT-29 or SW480 treated with RSL3, BAY 87-2243 alone or in combination treatment were detected using flow cytometry with C11-BODIPY 581/591 (FITC channel; excitation/emission: 488/510 nm). (G) MDA levels in cells subjected to the indicated treatment. (H) GSH levels in cells subjected to the indicated treatment. Results are shown as means ± SD. ∗*P < 0.05*; ∗∗*P < 0.01*; ∗∗∗*P < 0.001*; ∗∗∗∗*P < 0.0001*. P values were calculated by one-way ANOVA.

To determine whether pharmacological inhibition of HIF-1α can sensitize MSS CRC cells to ferroptosis-inducer RSL3, we treated cells with the HIF-1α inhibitor BAY 87-2243. This compound reduces HIF-1α protein level by inhibiting mitochondrial complex I activity, thereby suppressing the expression of HIF-1α target genes [32,33]. Combined treatment with BAY 87-2243 and RSL3 significantly reduced cell viability compared to monotherapy with either RSL3 or BAY 87-2243 alone in HT-29 or SW480 cells (Fig. 5A–B). Flow cytometric analysis revealed elevated DCFH-DA oxidation following combination treatment with BAY 87-2243 and RSL3 in HT-29 and SW480 cells (Fig. 5C–D). Consistently, lipid peroxidation accumulation was increased, confirming enhanced ferroptosis by combination treatment (Fig. 5E–F). Meanwhile, BAY 87-2243 also augmented RSL3-induced MDA production (Fig. 5G). As System x_c_^−^-GSH-GPX4 axis serves as a key ferroptosis defense mechanism by restraining lipid peroxidation [34], GSH levels were subsequently assessed. GSH was markedly depleted in HT-29 and SW480 cells exposed to the BAY 87-2243 and RSL3 combination (Fig. 5H). Similar results were observed with BAY 87-2243 and Erastin combination treatment. Cell viability was substantially reduced in the combination treatment group compared to monotherapy in HT-29 or SW480 cells (Supplementary Fig. S2A and S2B). The levels of DCFH-DA oxidation, lipid peroxidation, MDA were consistently elevated, while GSH synthesis was decreased in the BAY 87-2243 and Erastin combination treatment group compared to single-agent treatment (Supplementary Fig. S2C-S2H). These findings demonstrated that the HIF-1α inhibitor significantly increased the ferroptosis sensitivity in MSS CRC cells.

### 3.6 HIF-1α inhibitor enhanced RSL3-mediated antitumor effects and reduced liver metastasis

**Fig. 6:**
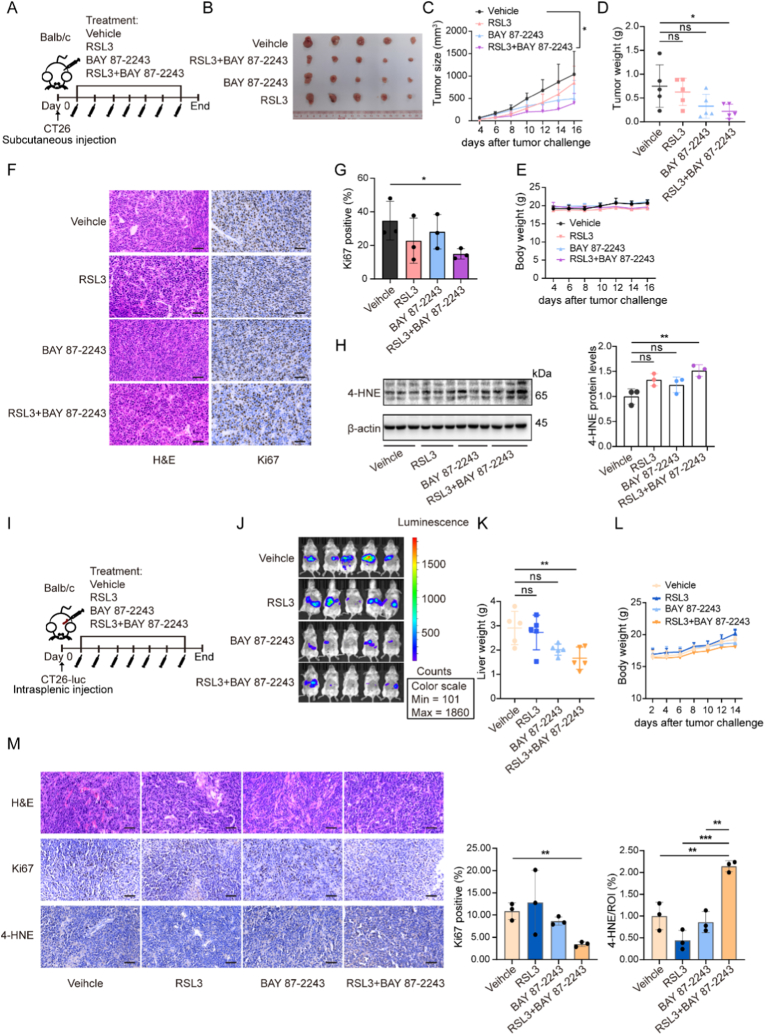
HIF-1α inhibition attenuated tumor growth and liver metastasis *in vivo*. (A) Schematic diagram of the mouse model subcutaneously implanted with CT26 cells into balb/c mice. Mice were randomized to receive vehicle, RSL3 (80 mg/kg, intraperitoneal injection, three times per week), BAY 87-2243 (3 mg/kg, oral gavage, daily), or combination treatment when tumor size reached 50-80 mm^3^. Control mice were treated with the vehicle. (B) Photograph of tumors at experimental endpoint. (C) The tumor growth curve was shown. Tumor size was monitored by micrometer caliper measurement. *P* values were calculated by two-way ANOVA between the indicated groups. ∗*P* < 0.05. (D and E) Tumor weight and body weight were measured at the experimental endpoint, respectively. *P* values were calculated by unpaired Student's *t*-test. ∗*P* < 0.05; ns: not significant. (F) Representative images of H&E staining (left) and immunohistochemical staining (right). Scale bar: 50 μm. (G) Quantification of positive Ki67 cells in each group. *P* value was calculated by unpaired Student's *t*-test. ∗*P* < 0.05. (H) 4-HNE expression in tumor tissues by western blotting. *P* values were calculated by unpaired Student's *t*-test. ∗∗*P* < 0.01; ns: not significant. (I) Schematic diagram of liver metastatic model by intrasplenic injection with CT26. (J-L) Bioluminescent images (J), liver weight (K), and body weight (L) in the liver metastatic model at endpoint. *P* values were calculated by unpaired Student's *t*-test. ∗∗*P* < 0.01; ns: not significant. (M) Representative images of immunostaining in the liver metastatic model at the endpoint. Scale bar: 50 μm. *P* values were calculated by unpaired Student's *t*-test. ∗∗*P* < 0.01; ∗∗∗*P* < 0.001.

To evaluate the therapeutic potential of BAY 87-2243 and RSL3 combination *in vivo*, we established subcutaneous tumor models using CT26 cells. Following tumor establishment, mice were randomized to receive vehicle, RSL3, BAY 87-2243, or the combination of RSL3 plus BAY 87-2243 (Fig. 6A). As anticipated, the combination of BAY 87-2243 and RSL3 demonstrated the most pronounced tumor growth inhibition compared to all other treatment groups (Fig. 6B-E). Immunohistochemical analysis revealed reduced Ki67-positive staining following combination treatment, indicating decreased cellular proliferation (Fig. 6F–G). The expression of 4-Hydroxynonenal (4-HNE), a notable marker for ferroptosis, was significantly increased after combination therapy (Fig. 6H).

Given that Liver metastasis is a primary pattern for MSS CRC and contributes significantly to recurrence in patients with advanced-stage disease, we investigated the effect of HIF-1α inhibition on metastatic progression. Based on our earlier findings that RSL3 pretreatment enhanced HIF-1α activation and self-renewal capacity (Fig. 3B-E), we hypothesized that inhibition of HIF-1α might exhibit a superior anti-metastasis effect *in vivo*. After intrasplenic injection with CT26, mice were administered either RSL3, BAY 87-2243 alone, or their combination (Fig. 6I). As expected, we observed significantly reduced liver metastases in the combination treatment group, accompanied by decreased liver weight (Fig. 6J-L). Immunohistochemical staining results confirmed a lower percentage of Ki67^+^-proliferative cells in the combination treatment group, while increased 4-HNE expression led to a reduction of hepatic metastases (Fig. 6M).

### 3.7 HIF-1α inhibitor promoted CD8^+^ T cells and CD86^+^ macrophages infiltration and attenuated tumor progression

**Fig. 7:**
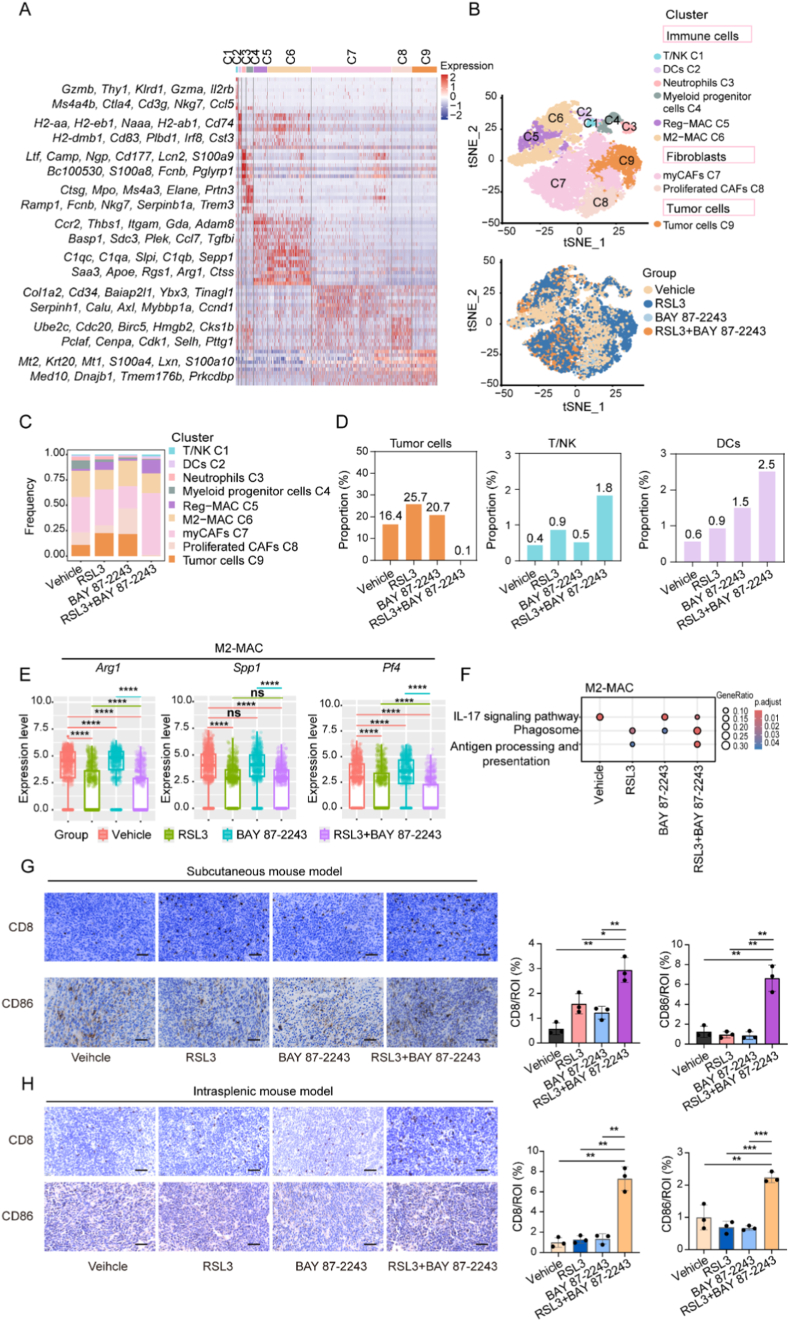
Single-cell sequencing illustrated that targeting HIF-1α improved RSL3 therapeutic efficiency through reversing the tumor immune suppression microenvironment. (A) Gene expression heatmap of different populations detected in the liver metastatic section from all treatment groups. (B) t-SNE visualization of all populations (up) and each treatment group (down). DCs: dendritic cells; Reg-MAC: regulated macrophage; M2-MAC: M2-like macrophage; myCAFs: myofibroblastic cancer-associated fibroblasts. (C) Frequency of each cluster across all groups. (D) The proportion of the indicated cluster in all treatment groups. (E) The distribution of selected genes was analyzed in the M2-MAC cluster by Student's *t*-test. ∗∗∗∗*P* < 0.0001; ns: not significant. (F) Kyoto Encyclopedia of Genes and Genomes (KEGG) analysis of M2-MAC. (G) Representative images of immunohistochemical staining in tumor tissue from the subcutaneous mouse model. *P* values were calculated by unpaired Student's *t*-test. ∗*P* < 0.05; ∗∗*P* < 0.01. (H) Representative images of immunohistochemical staining in tumor tissue from the liver metastasis mouse model. *P* values were calculated by unpaired Student's *t*-test. ∗∗*P* < 0.01; ∗∗∗*P* < 0.001.

MSS CRC displays immunologically ‘cold’ TME, limiting therapeutic efficacy. Recent studies show that ferroptosis induction enhances the efficacy of antitumor effects by triggering immune responses [35,36,37]. To comprehensively characterize alterations in the TME after drug treatment, liver metastasis tissues from CT26 intrasplenic transplantation mice were harvested for single-cell RNA sequencing analysis. The cells were clustered using t-SNE integrated map and subsequently annotated based on cell-type-specific marker gene expression (Fig. 7A–B). Distinct populations of tumor cells, cancer-associated fibroblasts, and immune cells were identified from liver metastasis lesion (Fig. 7B). Cell population proportions across treatment groups revealed a reduction in the percentage of tumor cells in the combination treatment group compared to the solvent control, indicating effective suppression of tumor cells within the metastatic lesion (Fig. 7C–D). Importantly, HIF-1α inhibition combined with RSL3 treatment increased the proportions of T/NK cells and dendritic cells (DCs) (Fig. 7C–D). M2-like macrophages (M2-MAC) were further authenticated through GSEA analysis. Regulated macrophages (Reg-MAC) enriched interferon response, prominent TNF signaling activity and IL2 pathway activation while reducing in M2-MAC (Supplementary Fig. S3). HIF-1α inhibition combined with RSL3 treatment manifestly decreased the expression of M2-MAC marker genes, including Arg*1*, *Spp1* and *Pf4* (Fig. 7E). Kyoto Encyclopedia of Genes and Genomes (KEGG) analysis also exhibited enhanced phagocytic capacity and antigen processing and presentation activities in M2-MAC (Fig. 7F). These findings suggested that HIF-1α inhibition enhanced RSL3 sensitivity by remodeling the tumor immune microenvironment.

To validate these observations, immunohistochemical staining was performed on tumor sections from both subcutaneous and liver metastasis mouse models. HIF-1α inhibition combined with RSL3 treatment significantly increased CD8^+^ T cell infiltration and elevated CD86 expression (a M1 macrophage marker) in the subcutaneous mouse model (Fig. 7G). Similar results were confirmed in an intrasplenic mouse model, which demonstrated enhanced CD8^+^ T cells and CD86^+^ macrophages infiltration in the combination treatment group (Fig. 7H). These data supported the capacity of HIF-1α blockade to sensitize tumor cells to ferroptosis by promoting the expansion of CD8^+^ T cells and CD86^+^ macrophages in MSS CRC.

### 3.8 Targeting HIF-1α improved anti-PD1 immunotherapy efficacy in MSS CRC

**Fig. 8:**
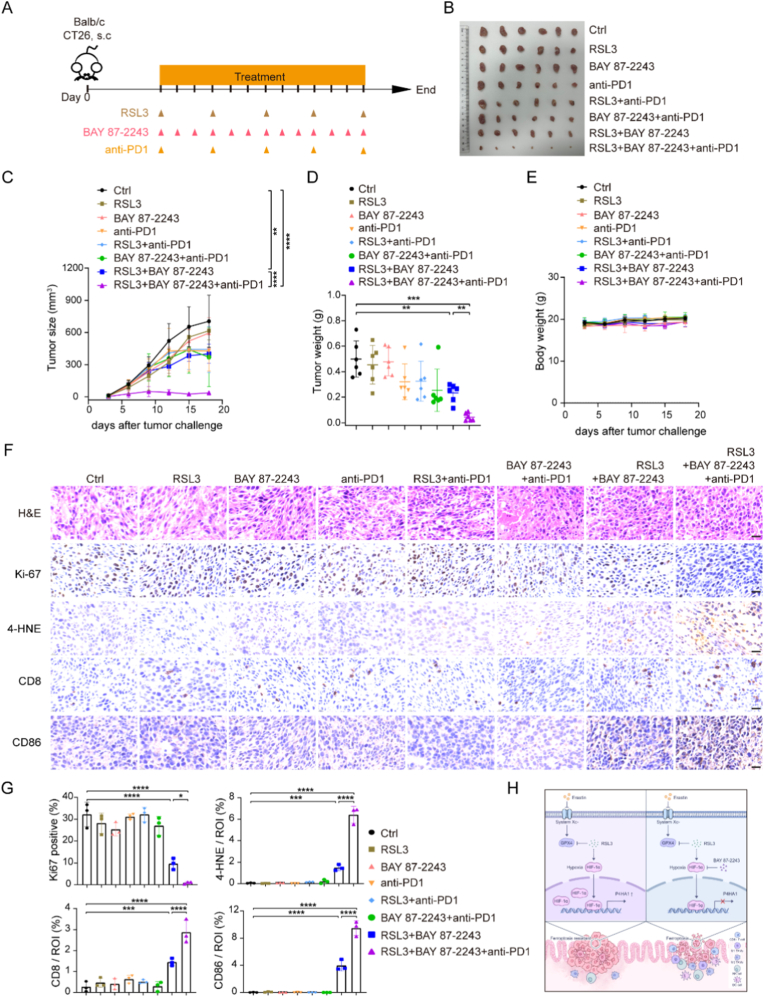
Targeting HIF-1α enhanced anti-PD1 therapeutic efficiency in MSS CRC. (A) Schematic of the experimental design for the mouse model. (B) Image of subcutaneous tumors from each treatment group. (C) Tumor growth curve across treatment groups. (D) Tumor weight at experiment endpoint. (E) Mouse body weight monitored throughout the experiment. (F) Representative H&E and immunohistochemical staining images of tumor sections. Scale bar: 20 μm. (G) Quantification of immunohistochemical staining results shown in (F). (H) Proposed working model illustrating HIF-1α-driven ferroptosis resistance in MSS CRC.

Given that MSS CRC is characterized by a low TMB and an immunosuppressive microenvironment, which shows minimal response to single-agent ICB, we hypothesized that targeting HIF-1α may potentiate the efficacy of anti-PD1 therapy. To test this, we orchestrated that ferroptosis induction, HIF-1α blockade combined with anti-PD1 in a mouse model (Fig. 8A). Anti-PD1 monotherapy demonstrated no effect on tumor control. While HIF-1α inhibition combined with RSL3 markedly suppressed tumor growth, and the addition of anti-PD1 to this regimen further improved therapeutic outcomes, demonstrating a synergistic effect (Fig. 8B-E). Consistent with this, HIF-1α blockade and RSL3 treatment in combination with anti-PD1 decreased cell proliferation and increased intratumoral ferroptosis (Fig. 8F–G). Further, immunohistochemical analysis revealed significant increases in CD8 and CD86 expression within tumor sections, indicating the elevated infiltration of CD8^+^ cytotoxic T cells and CD86^+^ macrophages (Fig. 8F–G). Collectively, these findings demonstrated that targeting HIF-1α enhanced the efficacy of anti-PD1 immunotherapy in MSS CRC, at least in part through the promotion of ferroptosis.

## 4 Discussion

Our study demonstrated that the hypoxic cell population within MSS CRC demonstrates significant ferroptosis resistance. Ferroptosis-resistant MSS CRC cells displayed increased malignancy and contributed to a highly immunosuppressive TME. The elevated HIF-1α-P4HA1 transcriptional regulatory axis is a key determinant of this resistance. Combined targeting of ferroptosis and HIF-1α effectively suppressed MSS CRC progression and metastasis by inducing ferroptosis-mediated cell death and reprogramming the immunologically ‘cold’ TME to a more immune-permissive ‘hot’ phenotype (Fig. 8H). This study identifies HIF-1α as a central regulator of ferroptosis resistance in MSS CRC. Although HIF-1α′s role in hypoxia adaptation and metabolic reprogramming is well established, its involvement in ferroptosis susceptibility provides a new mechanistic insight with important therapeutic implications for MSS CRC.

A distinct population of ferroptosis-resistant cells, characterized by simultaneous activation of hypoxia and glycolysis pathways, provides mechanistic insight into the failure of ferroptosis-based treatments. These cells exhibit increased tumorigenic potential and create a more immunosuppressive microenvironment, indicating that ferroptosis resistance may promote both local tumor growth and systemic metastasis. Mechanistic analysis identified P4HA1 as a direct transcriptional target of HIF-1α that mediates ferroptosis resistance. P4HA1, an enzyme involved in collagen hydroxylation, consumes α-ketoglutarate and produces succinate, which may alter TCA cycle flux and cellular redox balance. Additionally, P4HA1 is also implicated in the regulation of fatty acid metabolism and susceptibility to lipid peroxidation [38]. Hence, the HIF-1α-P4HA1 axis represents an attractive therapeutic target, as disrupting this pathway could simultaneously enhance ferroptosis sensitivity and disrupt tumor-supportive metabolic programs. Our findings expand the growing understanding of how metabolic reprogramming influences therapy resistance in cancer. The concept that metabolic adaptations can simultaneously promote survival, growth, and treatment resistance suggests that targeting metabolic vulnerabilities may be more effective than approaches focused solely on proliferation or apoptosis pathways.

Identifying HIF-1α as a druggable target to overcome ferroptosis resistance has immediate clinical relevance. By combining RSL3 with HIF-1α inhibition, we observed a significant immunological shift from M2 to M1-like (CD86^+^) macrophages and a marked increase in CD8^+^ T cell infiltration within the tumor microenvironment. These findings suggest that ferroptosis induction, when decoupled from its resistance mechanism, acts as a form of immunogenic cell death (ICD). This dual action of direct ferroptosis-mediated killing and TME reprogramming successfully converted non-responsive MSS tumors into responders to anti-PD1 therapy. Such synergy mirrors recent successes in other therapeutic recalcitrant cancers, such as metastatic gastric cancer, where combining ferroptosis induction with CAR-T therapy produced notable responses [39]. Collectively, our results establish a compelling therapeutic rationale for a ‘triple-combination’ regimen incorporating a ferroptosis inducer, a HIF-1α inhibitor and an immune checkpoint blockade agent as a strategy to overcome the current therapeutic plateau in MSS CRC management.

## 5 Conclusion

In summary, this study demonstrates that HIF-1α plays a critical role in mediating ferroptosis resistance in MSS CRC. Elevated HIF-1α expression in MSS CRC patients correlates with poor prognosis. Single-cell sequencing identified a distinct HIF-1α-regulated cell population with concurrent activation of hypoxia and glycolysis pathways, which is transcriptionally distinct and exhibits intrinsic ferroptosis resistance and enhanced malignant potential. Mechanistically, HIF-1α directly transcriptionally activates P4HA1, and the HIF-1α-P4HA1 signaling axis was found to be a key determinant of both ferroptosis resistance and tumor progression in MSS CRC. Pharmacological inhibition of HIF-1α significantly increased ferroptosis sensitivity in MSS CRC cells in both *in vitro* and *in vivo* models. Importantly, the combination of RSL3 and HIF-1α inhibition effectively reprogrammed the immunosuppressive MSS CRC microenvironment from an ‘immune-cold’ to an ‘immune-hot’ state, characterized by increased infiltration of CD8^+^ cytotoxic T cells and CD86^+^ macrophages and a reduction in M2-like immunosuppressive macrophages. This shift was sufficient to sensitize previously refractory MSS CRC tumors to anti-PD1 immunotherapy. These findings provide a robust mechanistic rationale for clinical trials evaluating the combination of hypoxia-targeting agents and ferroptosis inducers to enhance the efficacy of cancer immunotherapy in patients with MSS CRC.

## CRediT authorship contribution statement

**Zhiying Yang:** Data curation, Formal analysis, Investigation, Methodology, Project administration, Software, Writing – original draft, Writing – review & editing. **Rui Ma:** Data curation, Formal analysis, Funding acquisition, Methodology. **Weili Wu:** Data curation, Formal analysis, Methodology. **Ying Shi:** Data curation, Formal analysis. **You Chen:** Methodology. **Xiaotong Luo:** Data curation, Formal analysis. **Kai Li:** Investigation. **Liangcai Wu:** Conceptualization, Funding acquisition. **Bo Wang:** Conceptualization, Funding acquisition, Supervision. **Boyu Zhang:** Conceptualization, Data curation, Formal analysis, Funding acquisition, Investigation, Methodology, Project administration, Software, Validation, Writing – review & editing. **Ping Yuan:** Conceptualization, Data curation, Formal analysis, Funding acquisition, Project administration, Resources, Software, Supervision, Validation, Visualization, Writing – original draft, Writing – review & editing.

## Declaration of competing interest

The authors declare that they have no known competing financial interests or personal relationships that could have appeared to influence the work reported in this paper.

## Data Availability

The scRNA-seq raw data reported in this study have been deposited in Genome Sequence Archive (GSA) under accession numbers HRA013855 and CRA031401. All other data supporting the findings of this study are available from the corresponding author upon reasonable request.

## Abbreviations

ANKZF1: ankyrin repeat and zinc finger peptidyl TRNA hydrolase 1; DDIT4: DNA damage inducible transcript 4; ENO1: enolase 1; ENO2: enolase 2; FAM162A: family with sequence similarity 162 member A; LDHA: lactate dehydrogenase A; MIF: macrophage migration inhibitory factor; P4HA2: prolyl 4-hydroxylase subunit alpha 2; PGK1: phosphoglycerate kinase 1; Arg1: arginase 1; Spp1: secreted phosphoprotein 1; Pf4: platelet factor 4.
