# Supplementary material for: Targeting HIF-1α promotes ferroptosis and boosts antitumor immunity in MSS colorectal cancer
Source: Redox Biol. 2026 Apr 1;93:104151. doi: 10.1016/j.redox.2026.104151 (PMC13090715; doi:10.1016/j.redox.2026.104151)
Supplement: Multimedia component 2 [file mmc2.docx]

**Supplementary figures**


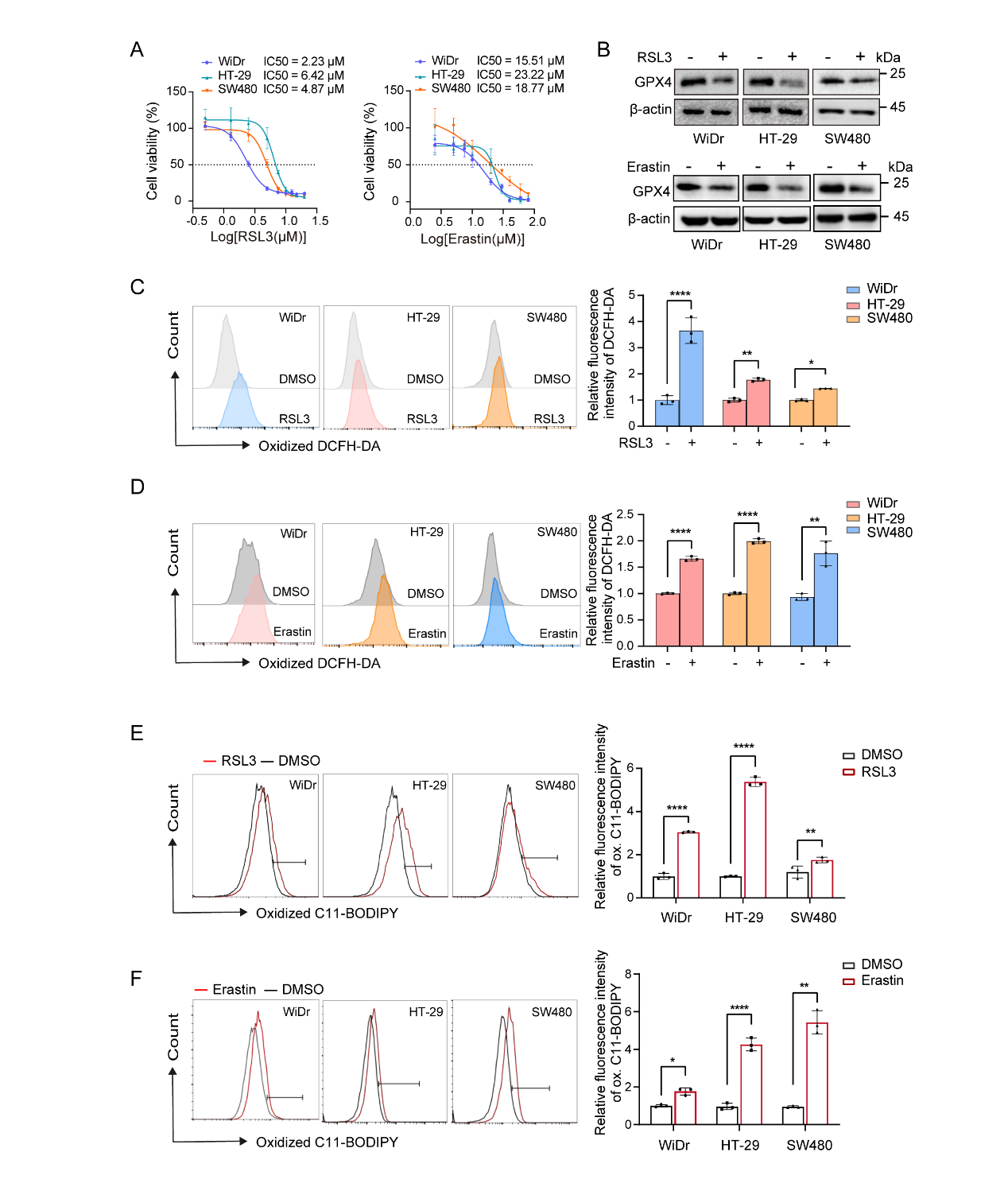


Fig. S1. Ferroptosis was induced by RSL3 and Erastin in MSS colorectal cancer cells. (A) Growth curves demonstrating dose-dependent effects of RSL3 or Erastin on cellular proliferation in WiDr, HT-29, or SW480 after 48-hour exposure. (B) WiDr, HT-29, and SW480 cells were treated with RSL3 or Erastin for 48 hours, followed by cell collection for western blot analysis. (C and D) After treating cells with RSL3 or Erastin for 48 hours, DCFH-DA oxidation was measured by flow cytometry. (E and F) Lipid peroxidation was assessed using C11-BODIPY 581/591. After 48-hour treatment with RSL3 or Erastin, oxidized C11-BODIPY was detected by flow cytometry in the FITC channel (excitation/emission: 488/510 nm), reflecting the level of lipid peroxidation. ox. C11-BODIPY: oxidized C11-BODIPY. Data are presented as means ± SD. **P < 0.05*; ***P < 0.01*; ****P < 0.001*; *****P < 0.0001*. P values were calculated by two-tailed t-test.


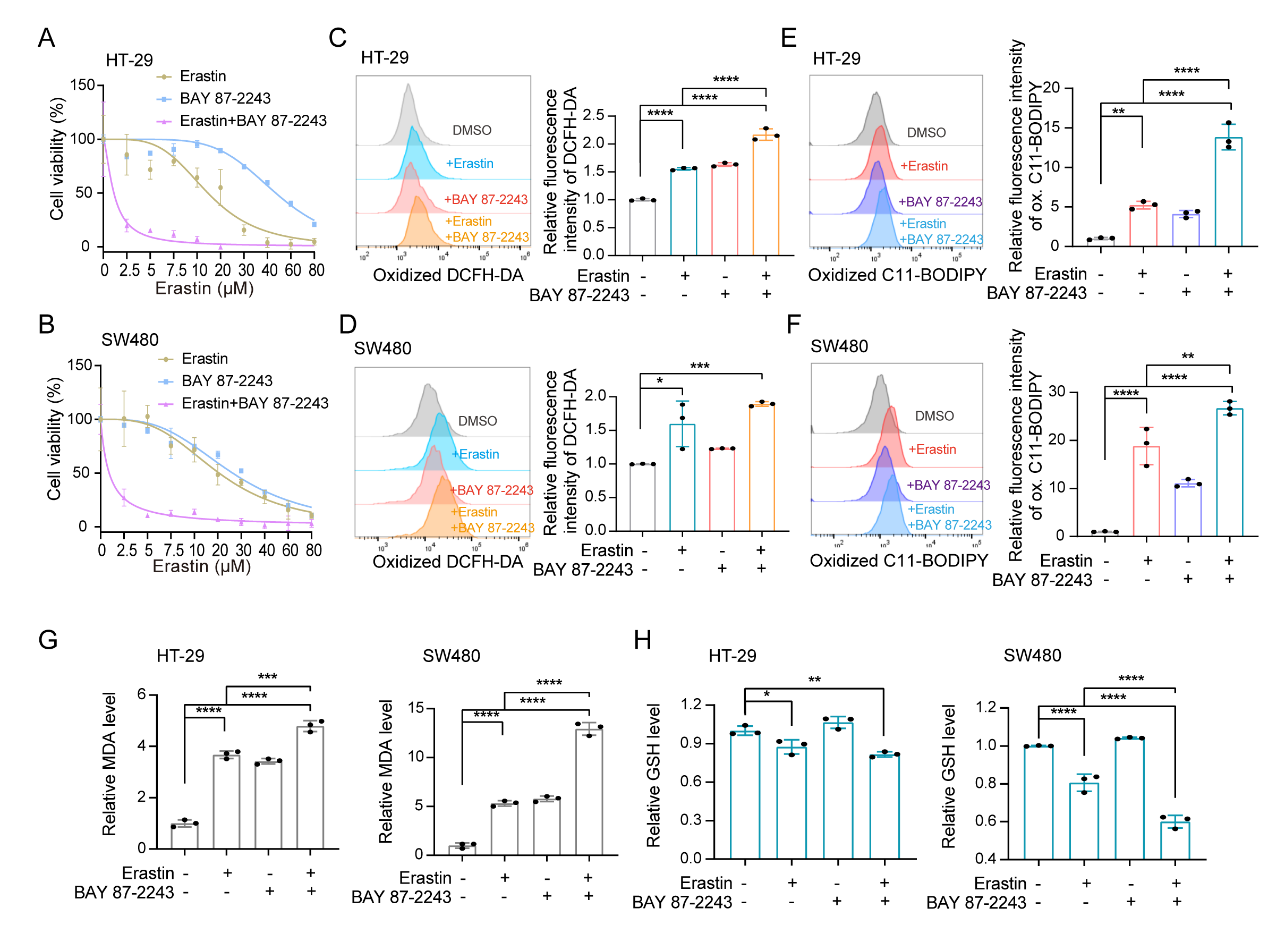


Fig. S2. HIF-1α inhibition enhanced Erastin sensitivity in MSS colorectal cancer cells. (A and B) Cell viability of HT-29 or SW480 treated with Erastin, BAY 87-2243 alone or combination treatment. (C and D) DCFH-DA oxidation in HT-29 or SW480 treated with Erastin, BAY 87-2243 alone or combination treatment was assessed by flow cytometry using the DCFH-DA probe. (E and F) Lipid peroxidation levels in HT-29 or SW480 treated with Erastin, BAY 87-2243 alone or combination treatment were evaluated by flow cytometry using the C11-BODIPY 581/591 probe. (G) MDA levels were assessed in HT-29 or SW480 treated with the indicated treatment. (H) HT-29 and SW480 cells were harvested for GSH analysis after treatment. Results are shown as means ± SD. **P* < 0.05; ***P* < 0.01; ****P* < 0.001; *****P* < 0.0001. *P* values were calculated by one-way ANOVA.


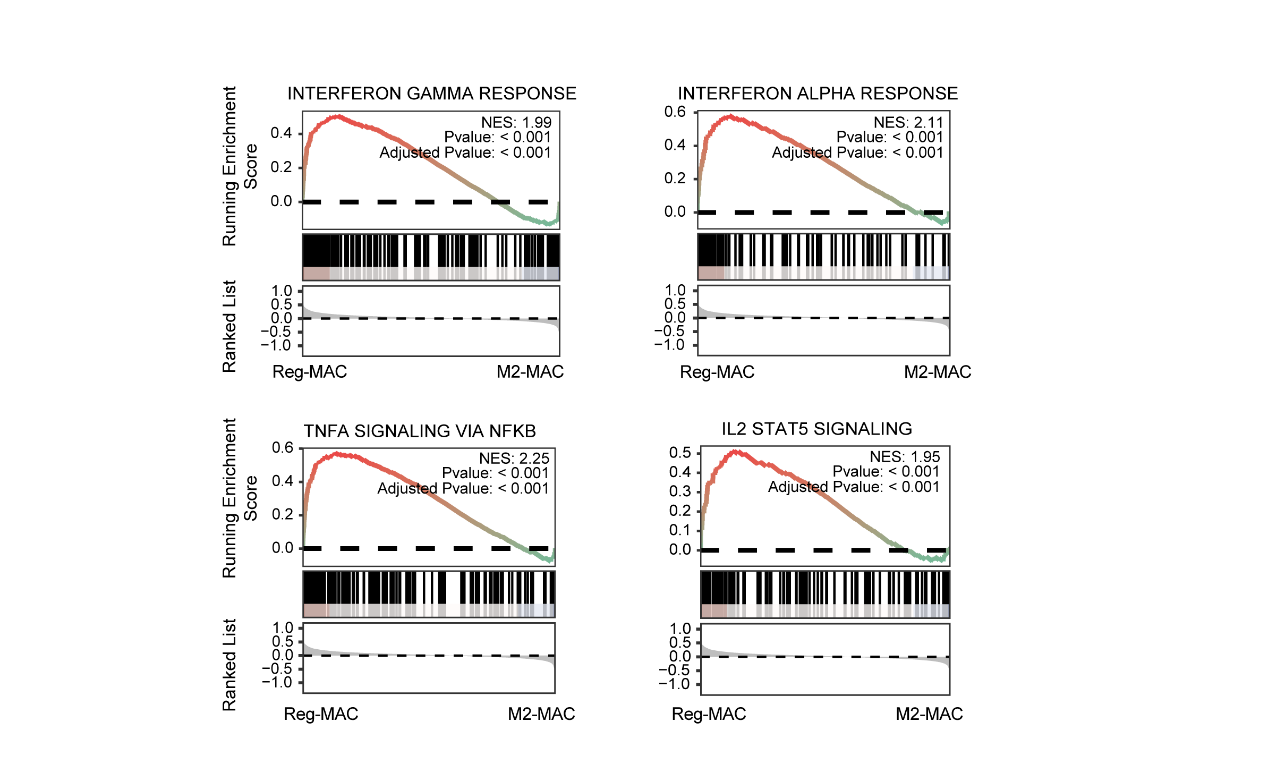


Fig. S3. GSEA analysis of signaling pathways between regulatory macrophages (Reg-MAC) and M2-liked macrophages (M2-MAC).
