# Supplementary figures and images for: Targeting HIF-1α promotes ferroptosis and boosts antitumor immunity in MSS colorectal cancer
Source: Redox Biol. 2026 Apr 1;93:104151. doi: 10.1016/j.redox.2026.104151 (PMC13090715; doi:10.1016/j.redox.2026.104151)

Raw data of western blot assay in this research


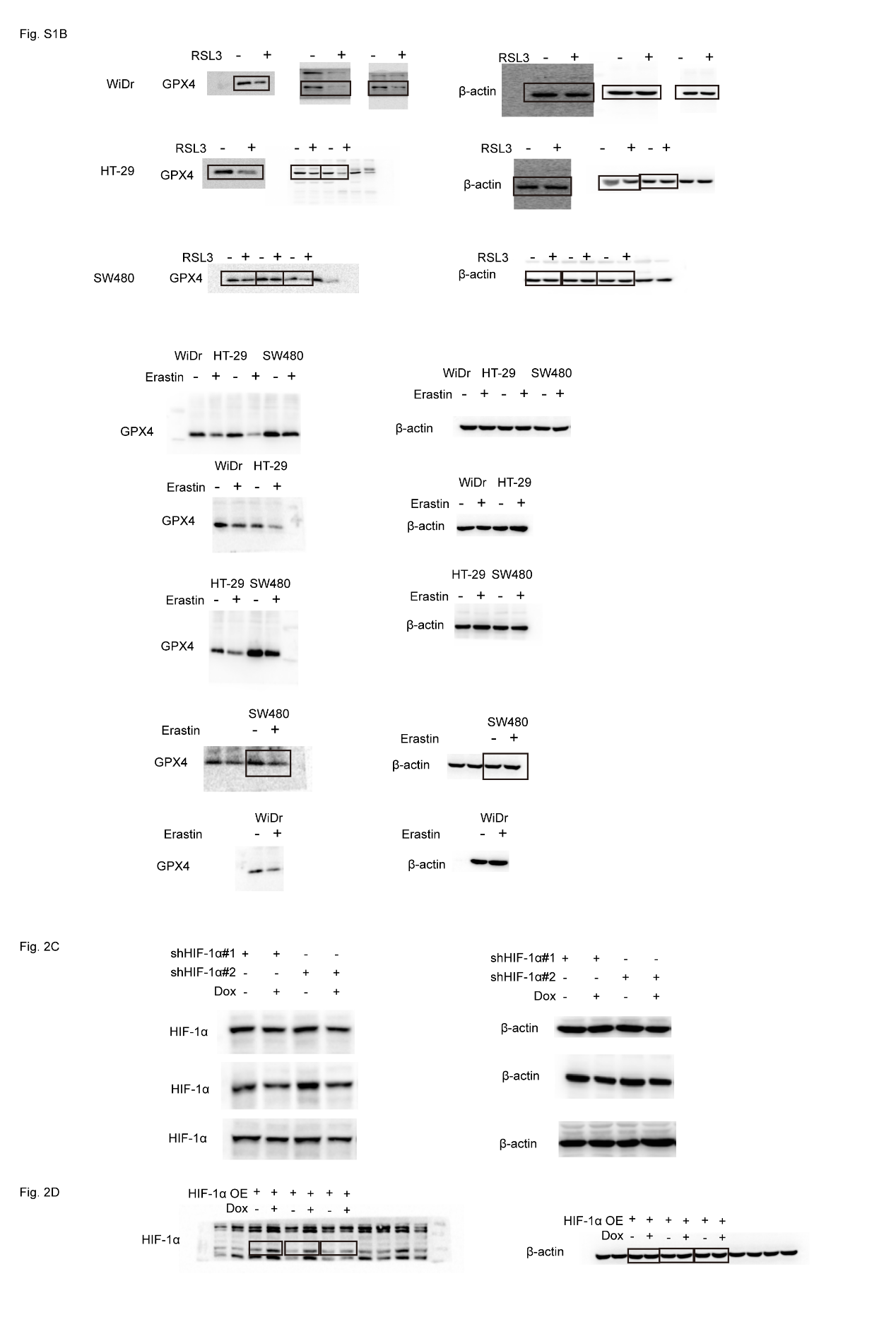

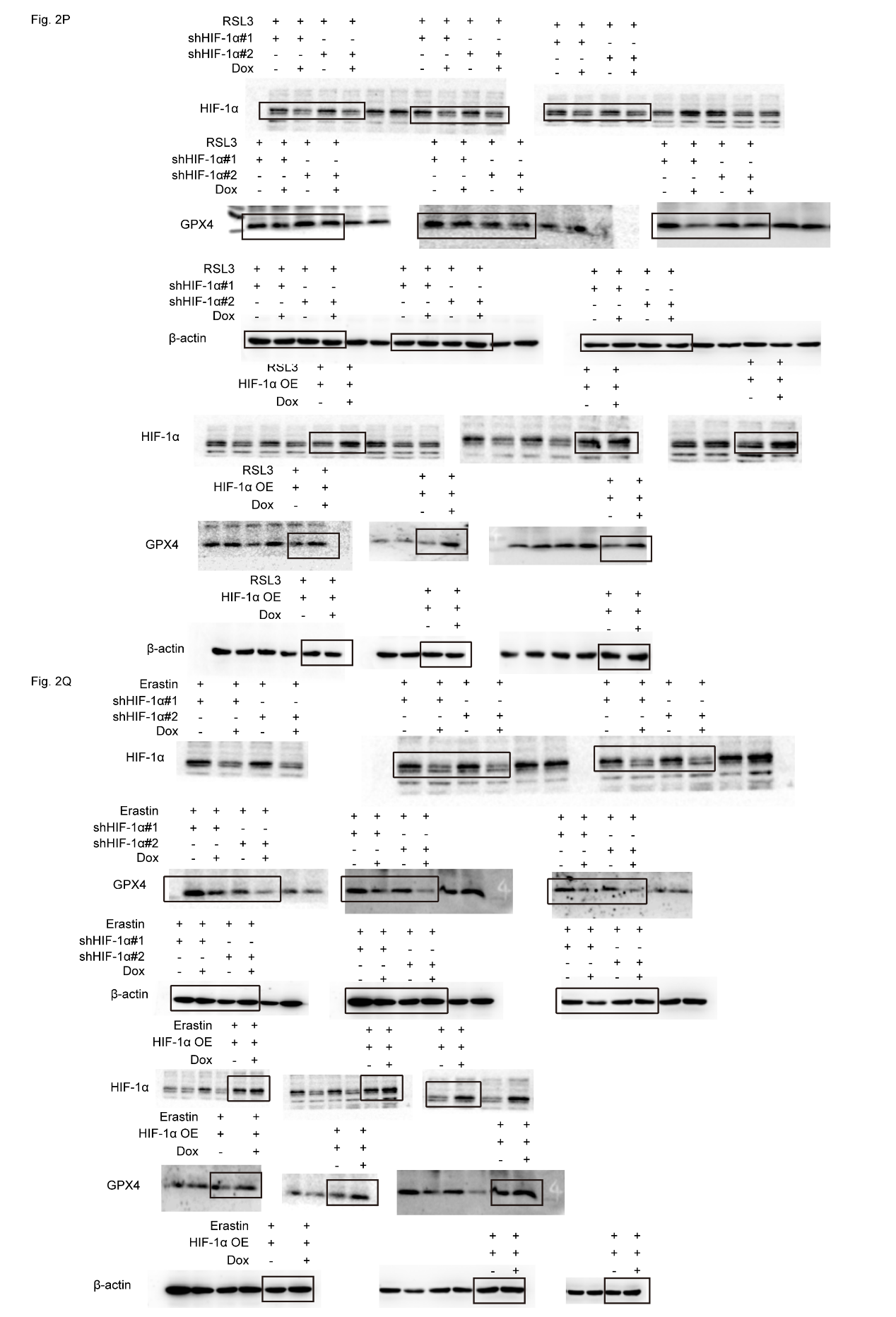

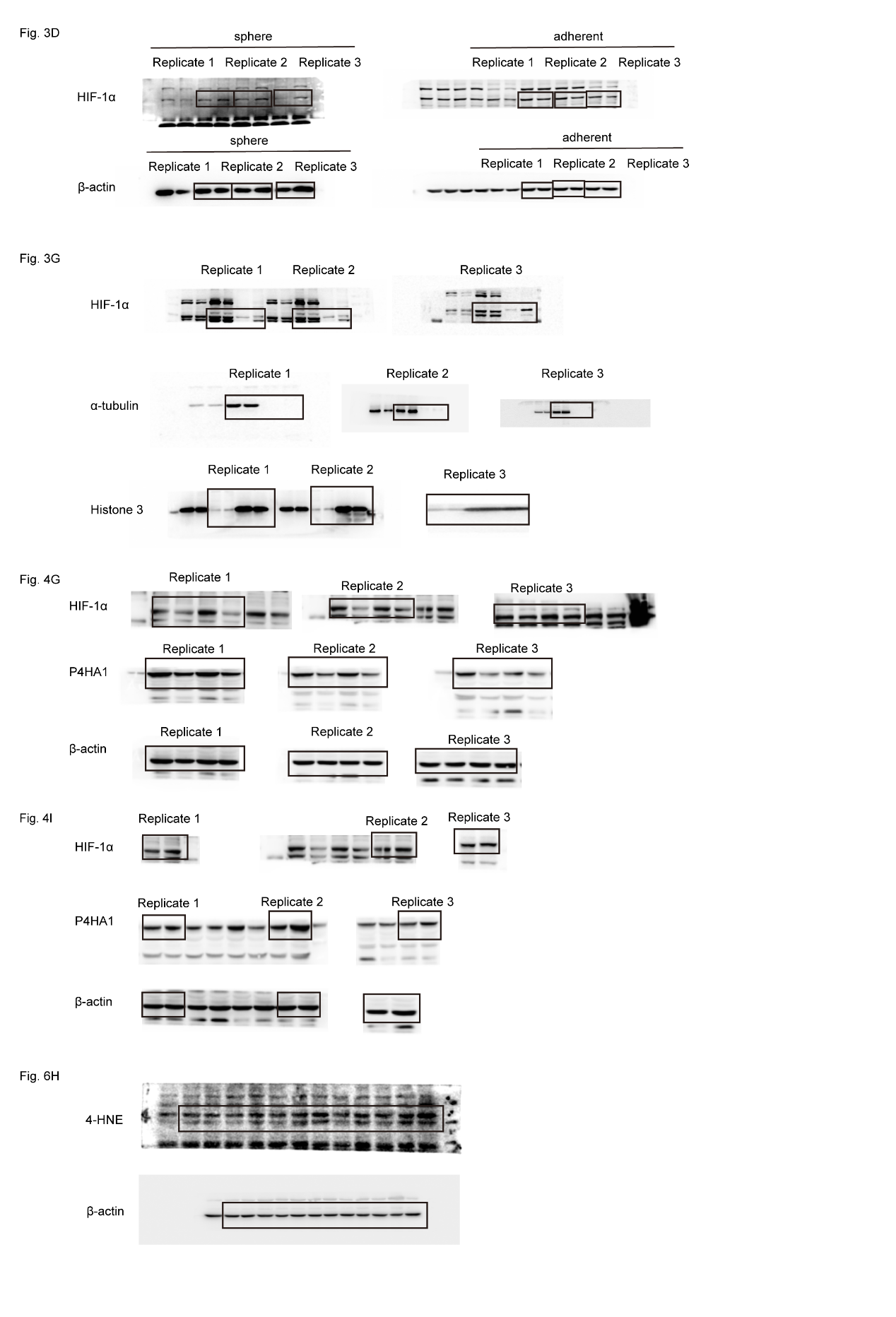

Supplement: Multimedia component 3 [file mmc3.docx]
